# Catalytic Site Cysteines of Thiol Enzyme: Sulfurtransferases

**DOI:** 10.4061/2011/709404

**Published:** 2010-12-28

**Authors:** Noriyuki Nagahara

**Affiliations:** Department of Environmental Medicine, Nippon Medical School, 1-1-5 Sendagi Bunkyo-ku, Tokyo 113-8602, Japan

## Abstract

Thiol enzymes have single- or double-catalytic site cysteine residues and are redox active. Oxidoreductases and isomerases contain double-catalytic site cysteine residues, which are oxidized to a disulfide via a sulfenyl intermediate and reduced to a thiol or a thiolate. The redox changes of these enzymes are involved in their catalytic processes. On the other hand, transferases, and also some phosphatases and hydrolases, have a single-catalytic site cysteine residue. The cysteines are redox active, but their sulfenyl forms, which are inactive, are not well explained biologically. In particular, oxidized forms of sulfurtransferases, such as mercaptopyruvate sulfurtransferase and thiosulfate sulfurtransferase, are not reduced by reduced glutathione but by reduced thioredoxin. This paper focuses on why the catalytic site cysteine of sulfurtransferase is redox active.

## 1. Introduction

 Cysteine residues in proteins maintain the protein conformation, coordinate metal(s), and regulate protein function [1–3]. Enzymes with catalytic site cysteines (Table 1) [4–42] have critical roles in biologic processes such as cell cycle regulation, apoptosis, and signal transduction [43].

 A cysteine residue that easily accepts and donates (an) electron(s) is referred to as a redox-active cysteine, and has a lower p*K*_a_ value than an unperturbed cysteine residue. Clairborne and colleagues extensively and successfully studied redox changes of cysteine residues and reviewed the biologic importance of redox-active cysteine [44, 45]; a redox-active cysteine is generally a thiolate at physiologic pH and is easily oxidized to a sulfenic acid. Cysteine-related enzymes are generally inhibited by mild oxidation and are reversibly reduced by thioredoxin or glutathione. The sulfenyl form is a reaction intermediate for peroxiredoxin to form disulfide [46] or protein tyrosine phosphatase 1B to form sulfenyl amide [47, 48]. 

 The sulfenyl form is further oxidized to the sulfinyl form and/or sulfonyl form. It is noteworthy that cysteine sulfinate desulfinase catalyzes the desulfination of cysteine sulfinic acid [49, 50], which is not a reversible reaction. On the other hand, cysteine sulfinic acid reductase (sulfiredoxin) catalyzes the reduction of cysteine sulfinic acid [51, 52], although neither thioredoxin nor glutathione can reduce sulfinic acid. Thus, sulfination of cysteine residues is a reversible oxidative process under the conditions that cysteine sulfinic acid reductase can access the catalytic site cysteine of an enzyme. When the reductase cannot access the catalytic site cysteine, sulfination is as irreversible as sulfonation. Recent studies in redox biology indicate that sulfenic acid is a molecular switch [53].

## 2. The Catalytic Site Cysteine Residue of Sulfurtransferase Is Redox Active

 The catalytic site cysteine of a thiol enzyme is generally redox active: a cysteine residue with a low p*K*_a_ value easily accepts and donates (an) electron(s). The catalytic site cysteine is essential for oxidoreductase to form a (intramolecular) disulfide and/or sulfenyl intermediate, and its high reactivity of the nucleophilic cysteine is advantageous for the catalysis of transferase (desulfurase, phosphatase, and sulfurtransferase), hydrolase (cysteine protease), and isomerase (protein disulfide isomerase) (Table 1).

 The effects of perturbing the p*K*_a_ of a cysteine residue in a protein are not well explained. It is generally considered that a decrease in the p*K*_a_ of a cysteine residue is caused by positively charged groups of neighboring amino acid residues and/or strengthening of electrostatic interactions between the group and the sulfur atom due to an increase in the electron density of the sulfur atom of the cysteine residue. Further, hydrogen bonding stabilizes the proton-dissociated state of the cysteine residue to maintain the p*K*_a_ perturbation. Hol and colleagues proposed the interesting notion that the alpha-helix macropole in a protein structure contributes to lowering the p*K*_a_ of a cysteine residue [54, 55].

**Figure 1:**
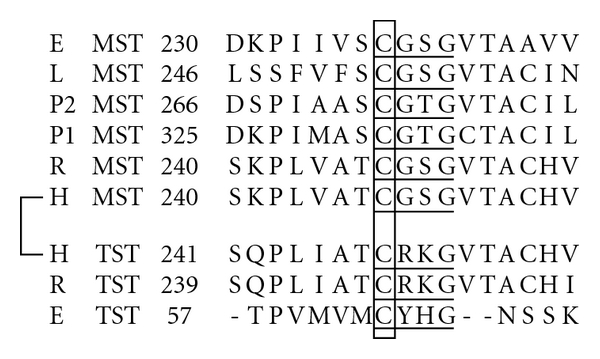
Comparison of the amino acid sequences around a catalytic site cysteine residue between MST and TST Sequence identity was analyzed using GENETYX (GENETYX CORPORATION). Box, a catalytic site. E, *E. coli* (D10496 for MST, NP_417883 for TST); H, *Homo sapiens* (BC009450 for MST, D87292 for TST); L. *Leishmania* (CAC85741); P1 and P2, *Arabidopsis thaliana* (AB032864 and AB032865 for MSTs); R, *Rattus norvegicus* (D50564 for MST, BC088449 for TST). Underlined amino acids, consensus sequences for MST or TST.

 Comparative studies of primary structures of sulfurtransferases (mercaptopyruvate sulfurtransferase [MST] and evolutionarily related rhodanese [TST] [22, 55, 56]) revealed that the consensus sequences around the catalytic cysteine of MST and TST are CG(S/T)G and C(R/Y)(K/H)G, respectively (Figure 1) [22, 55, 56].

**Figure 2:**
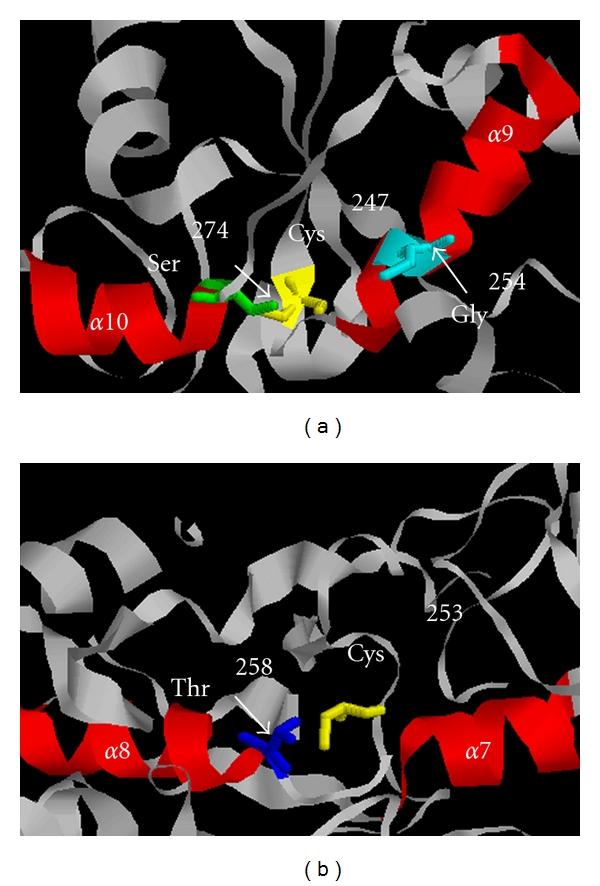
Model for the two *α*-helix dipoles of TST and MST, each structure is represented using RasMol. (a) bovine liver TST from 1DP2, red ribbon structure represents two helix-dipoles (*α*9 and *α*10) and ball-and-stick model in yellow represents a catalytic site Cys247. (b) *Leishmania major* MST from 1CKG red ribbon structure represents two helix-dipoles (*α*8 and *α*9), red ribbon structure represents two helix-dipoles (*α*8 and *α*9), and ball-and-stick model in yellow represents a catalytic site Cys253.

 The tertiary structures of MST and TST are persulfurated enzymes and stable catalytic intermediates (and also free-TST) [57–60]. In X-ray structural studies of bovine TST by Ploegman and colleagues [58–60] and Hol et al. [61], persulfide was stabilized by a ring of persulfide-stabilizing NH groups; Arg^248^, Lys^249^, Val^251^, and Thr^252^ (Figure 1) contributed to hydrogen bonding with an outer sulfur atom of a persulfide at the catalytic site Cys^247^, and in addition, Gly^254^ and Ser^274^ with the S*γ* of Cys^247^. Further, two helix-dipoles (*α*9 and *α*10) (Figure 2(a)) contribute to lowering the p*K*_a_ of the catalytic cysteine residue to approximately 6.5 [54, 62]. 

 Similar to TST, an X-ray structural study of *Leishmania major* persulfurated MST by Alphey et al. [57] revealed that Gly^254^, Ser^255^, Gly^256^, Val^257^, Thr^258^, and Ala^259^ (Figure 1) contribute to hydrogen binding with an outer sulfur atom of a persulfide at the catalytic site Cys^253^, and further, Thr^258^ with the S*γ* of Cys^253^. Two helix-dipoles (*α*8 and *α*9) (Figure 2(b)) also contribute to lowering the p*K*_a_ of the catalytic cysteine.

 The Cdc25 phosphatase family is a rhodanese superfamily [64, 65], and the catalytic subunit contains an alpha-helix macropole like MST and TST [66], which could contribute to lowering the p*K*_a_ of the catalytic cysteine. A member of the pyridoxal 5′-phosphate-dependent enzyme family, cysteine desulfurase (*E. coli* NifS CsdB), has an alpha-helix macropole like MST and TST [63, 67].

 In sulfurtransferases, alpha-helix macropoles surrounding a catalytic cysteine characterize the cysteine as redox active, indicating that hydrogen bonding between an outer sulfur atom of a persulfide at the catalytic site cysteine with surrounding amino acids is important for stabilizing catalytic intermediates.

## 3. Sulfenate Formation at a Catalytic Site in Sulfurtransferase

**Figure 3:**
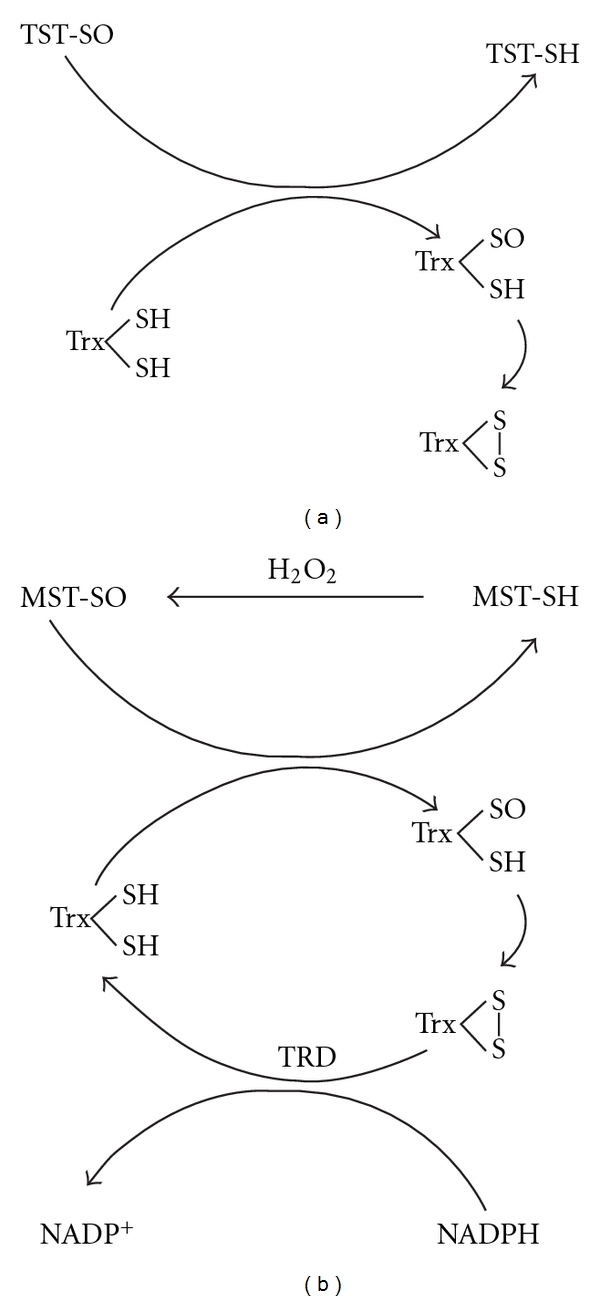
Thioredoxin oxidase activity of TST and thioredoxin peroxidase activity of MST, Proposed thioredoxin oxidase activity of TST (a) reported by Nandi and colleagues [63], which is same as thioredoxin peroxidase activity of MST (b) (from Figure  10 of Nagahara et al. Current Medical Chemistry 2009. 16: 4422). Trx: thioredoxin; TRD: thioredoxin reductase.

 When MST and TST are oxidized, catalytic site cysteines are reversibly sulfenated [23, 68] and are stable, probably due to hydrogen bonding. Sulfenyl TST was confirmed by the observation of thioredoxin oxidase activity and was reduced by reduced thioredoxin (Figure 3(a)) [68]. On the other hand, sulfenyl MST was confirmed by the observation of thioredoxin peroxidase activity (Figure 3(b)) and mass spectrometric data, and was reduced not by reduced glutathione but rather by reduced thioredoxin [23]. These findings indicate that the half-redox potential of sulfenate is lower than that of glutathione and higher than that of thioredoxin (“low redox potential sulfenate” [23]). The redox potential of the cysteine residue is pH-dependent due to pH-dependent perturbation of the electric field strength surrounding the cysteine residue via interactions of the cysteine residue with basic amino acids. In fact, the pH-dependent perturbation of the redox potential of the cysteine residue was demonstrated in the thioredoxin superfamily [69]. The active-site loop of TSTs contains two basic residues whereas no charged residues are observed in MSTs [65], suggesting that the electric field strength surrounding cysteine residue of mitochondrial TST is larger than that of MST. This hypothesis, however, has not been tested experimentally.

## 4. Possible Biologic Function of Catalytic SiteSulfenate of Sulfurtransferase

 Sulfenyl sulfurtransferase is neither a reaction intermediate nor an active form whereas the sulfenyl form is a reaction intermediate of a thiol-oxidoreductase. Therefore, the biologic relevance of a conversion between sulfenate and thiolate at a catalytic cysteine is not clear. There are two possibilities: first, the molecular feature was accidentally acquired during the molecular evolution of the thiol enzyme family, and second, some molecular entity, such as an antioxidant protein, has evolved under oxidizing atmospheric conditions.

MST and TST are widely distributed in eukaryotes and prokaryotes [56, 70], and in eukaryotic cells, MST is distributed in the cytoplasm, mitochondria, and in chloroplasts (in plants) [71, 72]. On the other hand, TST distribution is restricted to the mitochondria and chloroplasts (in plants) [72–74]. Thus, both MST and TST are located in mitochondria and chloroplasts (in plants). Based on the minor catalytic contributions, the latter possibility is likely: MST and TST could locally serve as antioxidant proteins.

 Unlike sulfurtransferases, a sulfenyl amide is found at the catalytic site cysteine in protein tyrosine phosphatase IB in an unusual oxidized form. This enzyme is oxidized to form sulfenate at the catalytic site cysteine, and the S*γ* atom of the cysteine covalently binds to the main chain nitrogen atom of an adjacent serine to form sulfenyl amide [47, 48]. This sulfenyl amide enzyme is inactive. Reduced glutathione cleaves (reduces) the ring structure of sulfenyl amide to completely restore activity [44, 45]. The redox regulation of the enzymatic activity correlates with signal transduction [75–77] via the regulation of protein dephosphorylation [78–81]. 

 Cdc25C, a member of the phosphatase family, has two redox active cysteines (Cys^330^ and Cys^377^). Mild oxidation forms sulfenate at one of the two redoxactive cysteines (Cys^377^) resulting in the formation of an intramolecular disulfide between them, which produces an inactive form of the enzyme [17, 18]. Further, the oxidized form is reduced not by reduced glutathione but rather by reduced thioredoxin [18], meaning that the cdc25 family forms a low redox potential disulfide. The redox regulation of the enzymatic activity correlates with the regulation of the cell cycle via the regulation of protein dephosphorylation [17, 18]. Further oxidation forms sulfinate at Cys^377^, which is an inactivated form, resulting in degradation of the protein [17]. 

 The cysteine protease caspase, which regulates apoptosis, is also inactivated by mild oxidation, probably due to sulfenate formation at the catalytic site cysteine, and can be reduced by reduced glutathione *in vitro* [33]. Physiologic levels of glutathione, however, are unable to restore activity [33], and other cellular reductants such as thioredoxin have not been examined. The biologic importance of redox regulation of the caspase activity remains unknown.

## 5. Summary

Both MST and TST are localized in mitochondria and chloroplasts, and probably serve as antioxidant proteins.

The catalytic site cysteine residue of MST and TST is redox active, probably due to helix dipoles.

Stable and low redox sulfenate is formed at the catalytic site cysteine of MST and TST, and is reduced by thioredoxin.

## Figures and Tables

**Table 1:** Typical thiol enzymes.

		Enzyme	Oxidative inactivation
Classification		name defined as a thiol enzyme	
Oxidoreductase		Glutathione family [4]	Not defined
		Glutaredoxin family [5]	Not defined
		Glyceraldehyde-3-phosphate dehydrogenase [6]	Yes [7, 8]
		Peptide-methionine (S)-S-oxide reductase [9]	Not defined
		Peroxiredoxin [10, 11]	Yes [12]
		Sulphiredoxin [13]	Not defined
		Thioredoxin family [14]	Not defined
Transferase	Desulfurase	Cysteine desulfurase^1^ [15]	Not defined
	Phosphatase	Cdc^2^ 25 family [16]	Yes [17, 18]
		Protein-tyrosine phosphatases [19]	Yes [20]
	Sulfurtransferase	Mercaptopyruvate sulfurtransferase [21, 22]	Yes [23]
		Thiosulfate sulfurtransferase [24]	Yes [25, 26]
Hydrolase	Cysteine protease	Actinidain family [27]	Not defined
		Bromelain family [28]	Yes [29]
		Calpain family [30]	Yes [31]
		Caspase family [32]	Yes [33]
		Cathepsin family [34]	Yes [35]
		Chymopapain family [36]	Yes [37]
		Ficin family [38]	Not defined
		Mir1-CP^3^ [39]	Not defined
		Papain family [40]	Yes [41]
Isomerase		Protein disulfide isomerase [42]	Not defined

^1^pyridoxal 5′-phosphate-dependent enzyme

^2^cdc, cell division cycle

^3^Mir1-CP. Maize insect resistance-cysteine protease
